# Compliance to completion of sodium valproate annual risk acknowledgement form among women of child-bearing age prescribed sodium valproate in the intellectual disability (ID) services of an NHS trust

**DOI:** 10.1192/bjo.2021.291

**Published:** 2021-06-18

**Authors:** Victor Ohize, Deval Bagalkote

**Affiliations:** 1 Nottinghamshire Healthcare NHS Foundation Trust; 2 Honorary Consultant Psychiatrist, Nottinghamshire Healthcare NHS Foundation Trust

## Abstract

**Aims:**

To determine the proportion of women of child-bearing age prescribed SV who have the SV ARF filled.

**Background:**

In 2018, the Medicines and Healthcare products Regulatory Agency (MHRA) gave guidance regarding Sodium Valproate (SV) prescription. It acknowledged the significant risk of birth defects and developmental disorders in women of child-bearing age prescribed SV.

Consequently, the MHRA recommendation is that SV must not be used in females of child-bearing age unless: conditions of pregnancy prevention programme are met; other treatments are ineffective or not tolerated; and evidence of discussion of risks with patient or carer and annual review of the risks are documented. The evidences of the above criteria are expected to be documented in an Annual Risk Acknowledgement Form (ARF).

**Method:**

Retrospective study involving systematic search of Trust database to identify women with ID, aged 16–50 years prescribed SV from 2018 to 2019.

**Result:**

18 of 28 patients had ARF filled, a 64% compliance.

The main indications for SV prescription were epilepsy; challenging behaviour; and mood stabilization.

The distribution showed neurology and psychiatrist led prescription initiation equally distributed at 50%.

The ARF compliance was higher in the neurology group (93%) compared to 36% in psychiatrist group.

A review across the 5 ID teams (A,B,C,D and E) of the trust shows variable compliance to ARF compliance (17%,81%,100%,60%,0% respectively) with teams having higher proportion of neurology led SV prescription initiation also having higher proportion of ARF completion compliance (0%,55%,80%,80%,0% respectively).

**Conclusion:**

Conclusion / Recommendation

ARF compliance is below standard at 64%.

Despite the SV prescription being equally distributed between neurology led and psychiatry led, patients whose prescription of SV is neurology led (prescription indication as epilepsy) had better ARF compliance outcome (93%) compared with patients whose prescription is psychiatry led (prescription indication as challenging behaviour or mood stabilization) with 36% ARF compliance.

Organizational difference with dedicated epilepsy nurse in the ID service means patients with epilepsy had reviews of medication and compliance to MHRA guidance in completing the ARF.

There is need to increase doctors’ awareness to review ARF status during patients’ appointment. Information Technology design to flag up out of date ARF may be helpful.

The review of ARF may also flag up consideration of other alternatives: behavioural, psychological, functional and environmental interventions as well as alternative medications like Risperidone for challenging behaviours and other mood stabilizing options. This will minimize SV prescription, which is the original goal of the MHRA guidance.

